# The Diagnostic Puzzle of Papillary Thyroid Carcinoma Without Risk Factors

**DOI:** 10.7759/cureus.87978

**Published:** 2025-07-15

**Authors:** Varchasvi Meena, Nishant Gill

**Affiliations:** 1 Otolaryngology - Head and Neck Surgery, Government Medical College, Kota, Kota, IND

**Keywords:** bilateral cervical lymphadenopathy, central neck dissection, papillary thyroid carcinoma, radiation, radioactive iodine

## Abstract

Papillary thyroid carcinoma (PTC) is the most common thyroid malignancy in children, yet its diagnosis in pediatric patients can be delayed due to atypical presentations and a low index of clinical suspicion. We report the case of a 12-year-old girl who presented with slowly progressive bilateral neck swellings. There were no classical risk factors such as prior radiation exposure, family history, or symptoms of thyroid dysfunction. Clinical examination and imaging revealed bilateral cervical lymphadenopathy, while initial fine-needle aspiration cytology (FNAC) suggested a benign colloid nodule. However, contrast-enhanced CT of the neck raised suspicion for malignancy, prompting total thyroidectomy with bilateral modified radical neck dissection and central compartment clearance. Histopathological examination confirmed papillary thyroid carcinoma with bilateral lymph node metastases. This case highlights the diagnostic complexity and clinical variability of pediatric PTC, especially in the absence of risk factors. Pediatric thyroid carcinomas, while more likely to present with lymph node involvement and distant spread, generally carry an excellent prognosis with timely intervention. This case emphasizes the need for heightened clinical awareness and an aggressive diagnostic workup, even in seemingly low-risk presentations. Early diagnosis and surgical management, followed by appropriate surveillance, are crucial for ensuring favorable long-term outcomes in pediatric patients with thyroid carcinoma.

## Introduction

Papillary thyroid carcinoma (PTC) is the most common type of thyroid malignancy in both adults and children, accounting for approximately 85-90% of all pediatric thyroid cancers [1,2]. However, thyroid cancer remains rare in the pediatric population, constituting only 1.8% of all thyroid malignancies and less than 2% of all childhood cancers [3]. Among pediatric patients, PTC typically presents with more aggressive features, including multifocal intrathyroidal disease, cervical lymph node metastasis, and, occasionally, distant pulmonary spread [4].

Despite this aggressive presentation, outcomes in children are generally favorable, with high overall survival rates exceeding 95% [5]. Classical risk factors for pediatric PTC include prior radiation exposure, familial syndromes (e.g., familial adenomatous polyposis, Cowden syndrome), and certain genetic mutations such as RET/PTC rearrangements and B-type RAF kinase (BRAF) mutations [6]. However, a subset of pediatric patients may present without any identifiable risk factors, making early diagnosis more challenging.

In this context, we present the case of a 12-year-old girl who exhibited bilateral cervical lymphadenopathy but had no known predisposing factors for thyroid malignancy. Initial cytology suggested a benign colloid nodule (Bethesda II), further complicating the diagnostic process. Ultimately, imaging and clinical judgment led to surgical intervention, and histopathology confirmed papillary thyroid carcinoma with metastatic lymph node involvement.

This case is noteworthy for its atypical presentation and the absence of classic risk factors, underscoring the need for heightened clinical suspicion even in seemingly low-risk pediatric patients. To our knowledge, such cases remain underreported in current literature, particularly those in which initial cytology is falsely reassuring. By detailing the diagnostic course, surgical approach, and follow-up, this report aims to contribute to the limited but growing body of evidence on sporadic pediatric PTC and advocate for a more cautious approach to thyroid nodules in children.

## Case presentation

This case highlights the clinical complexity and diagnostic nuances of PTC in a pediatric patient. A 12-year-old girl presented with bilateral neck swellings for one year. There was no history of radiation exposure, family history of thyroid disorders, or systemic symptoms. Examination revealed multiple non-tender cervical lymph nodes. Ultrasound-guided fine-needle aspiration cytology (FNAC) from the thyroid suggested a colloid nodule or follicular neoplasm. Ultrasonography showed a heterogeneous thyroid gland with multiple hypoechoic nodules, the largest measuring 7 × 4.4 mm. A Thyroid Imaging Reporting and Data System (TI-RADS) IV lesion was noted in the isthmus. Multiple enlarged cervical lymph nodes were seen, the largest measuring 29 × 13 mm. Contrast-enhanced computed tomography (CT) of the neck revealed a heterogeneous, ill-defined lesion (12 × 13 × 15 mm) in the right thyroid lobe with necrosis and exophytic extension. Enlarged necrotic lymph nodes were observed bilaterally. Although the CT and ultrasound reports were reviewed in detail, the corresponding images are not included due to suboptimal clarity and patient privacy considerations. Routine laboratory investigations, including thyroid function tests and serum markers, were conducted. The results are shown in Table 1.

**Table 1:** Pre-operative laboratory investigations This table summarizes the patient’s key thyroid-related and metabolic laboratory parameters, including thyroid hormones, thyroglobulin, and serum calcium, with respective reference ranges and units.

Test	Result	Reference Range	Units
Thyroid-stimulating hormone (TSH)	0.02	0.3–4.5	mIU/L
Triiodothyronine (T3)	140	70–200	ng/dL
Thyroxine (T4)	11.5	4.6–12.0	μg/dL
Serum thyroglobulin (Tg)	3.2	<1.0	ng/mL
Anti-thyroglobulin antibody (TgAb)	22	<40	IU/mL
Serum calcium	9.2	8.5–10.5	mg/dL

**Figure 1:**
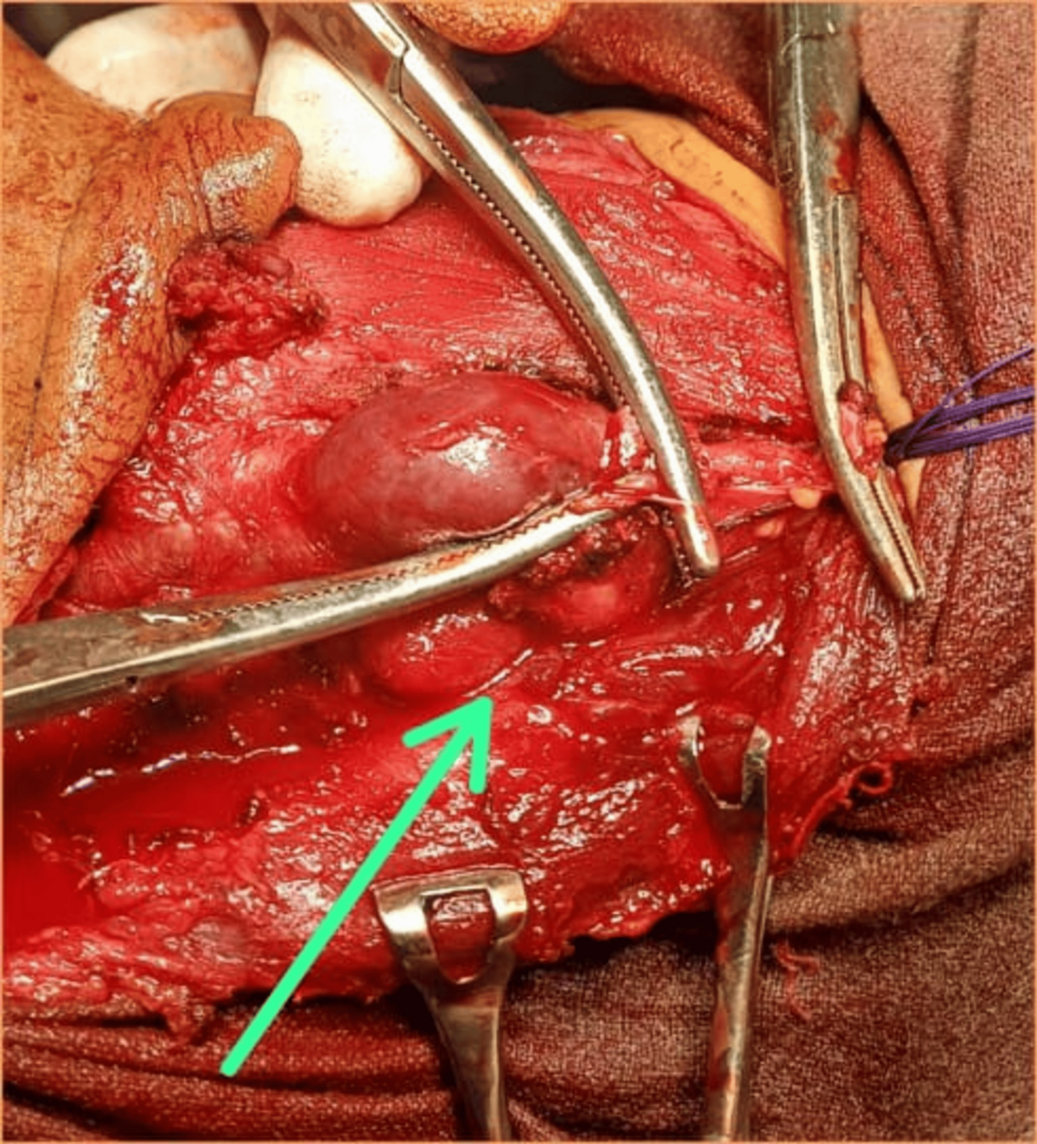
Intraoperative exposure of enlarged cervical lymph nodes Intraoperative photograph showing multiple enlarged bilateral cervical lymph nodes in levels II to V during neck dissection, consistent with nodal metastasis from papillary thyroid carcinoma.

Given these findings, the patient underwent total thyroidectomy with central compartment (level VI) and bilateral modified radical neck dissection. Postoperative recovery was uneventful, and she was started on levothyroxine therapy. An intraoperative photograph showing multiple enlarged cervical lymph nodes was taken (Figure 1).

**Figure 2:**
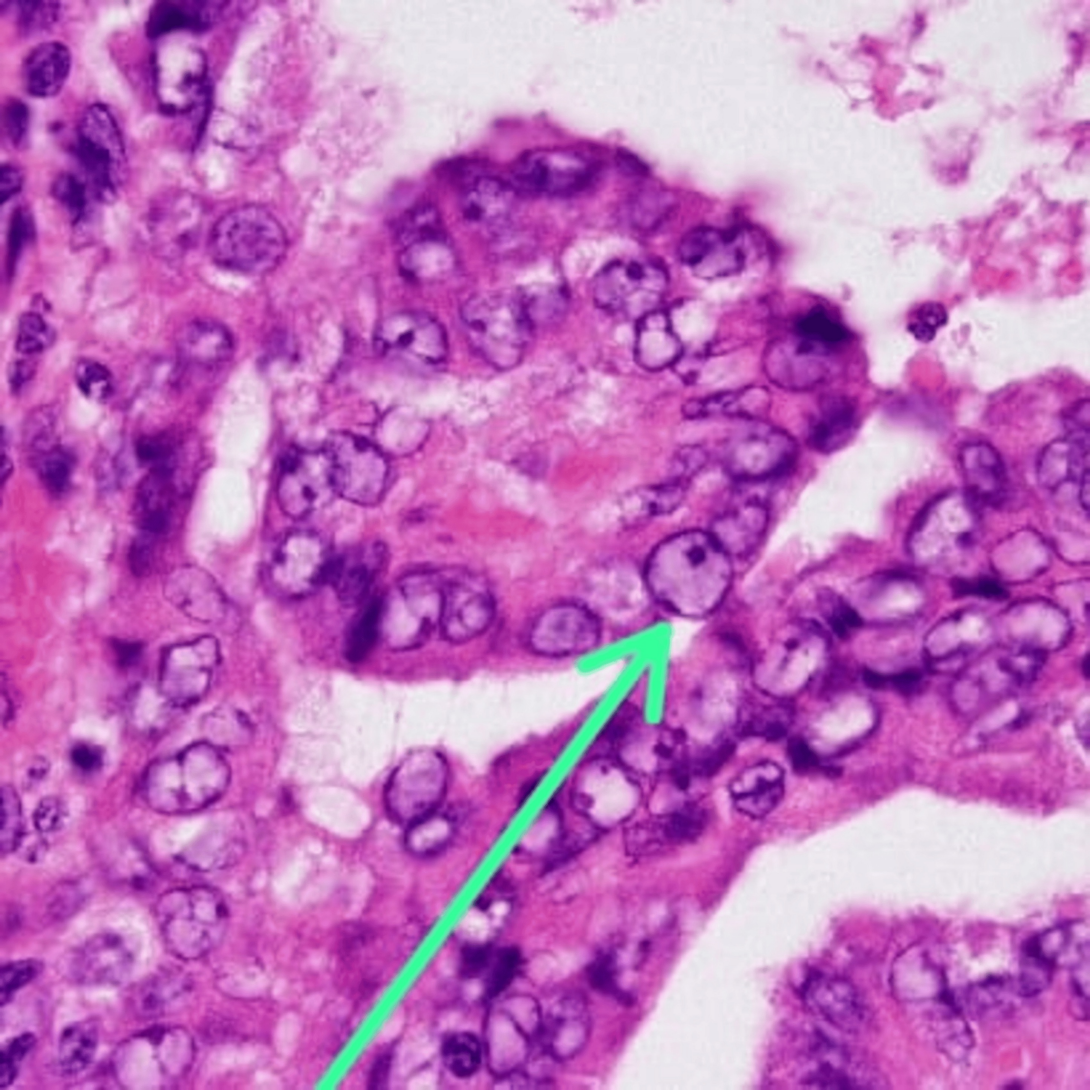
Histopathological section showing classic papillary thyroid carcinoma Histopathological examination (H&E stain, 40x) showing classic orphan Annie eye nuclei—characterized by optically clear, empty-appearing nuclei with central clearing.

Histopathological examination confirmed the diagnosis of papillary thyroid carcinoma. The primary thyroid lesion exhibited papillary structures with characteristic nuclear features, including overlapping, clearing, and nuclear grooves. These findings are depicted in Figure 2.

## Discussion

PTC accounts for approximately 90% of pediatric thyroid cancers and frequently presents as cervical lymphadenopathy [1]. Unlike adult cases, pediatric PTC often exhibits more extensive regional and distant spread, especially to the lungs, yet still carries an excellent prognosis with appropriate treatment. The most well-established risk factor remains radiation exposure, although familial syndromes, environmental factors, and gene mutations such as BRAF and rearranged during transfection (RET/PTC) also contribute [2]. Our patient, notably, had no identifiable risk factors. In such sporadic cases, accurate diagnosis hinges on careful clinical evaluation and imaging. Molecular testing has become increasingly relevant, offering prognostic insight and guiding therapeutic planning [3]. Although uncommon, occult papillary thyroid carcinoma may also present as a lateral cervical cyst, sometimes mimicking a benign lesion [4].

Surgical management is the cornerstone of treatment. Total thyroidectomy with central or lateral neck dissection is typically recommended when nodal involvement is evident. The American Thyroid Association (ATA) Pediatric Guidelines recommend stratifying patients using the American Joint Committee on Cancer (AJCC) Tumor, Node, Metastasis (TNM) system, followed by risk-based postoperative therapy [5]. Radioactive iodine (RAI) is usually reserved for intermediate or high-risk cases with nodal or distant disease.

In addition to surgical and adjuvant therapy, long-term surveillance is crucial. Pediatric patients require close surveillance with thyroglobulin monitoring, ultrasound imaging, and thyroid-stimulating hormone (TSH) suppression [6]. These strategies help detect recurrence early and maintain remission. Longitudinal studies support that while recurrence is more common in pediatric PTC, disease-specific mortality remains low, reinforcing the importance of timely and comprehensive treatment [7].

Several recent case reports have highlighted the occurrence of papillary thyroid carcinoma in children without identifiable risk factors. For instance, a 2021 case report by Zhang et al. [8] described a 10-year-old girl diagnosed with multifocal papillary thyroid carcinoma without any history of radiation exposure or family history. Similarly, Anand et al. [7] reported a 13-year-old boy with bilateral cervical lymphadenopathy and PTC, again in the absence of environmental or genetic predispositions. These reports reinforce the growing recognition that pediatric PTC can occur sporadically and may mimic benign pathology on initial evaluation, as in our case.

Differential diagnoses for pediatric neck swellings with thyroid involvement include benign colloid nodules, follicular neoplasms, reactive lymphadenopathy, tubercular lymphadenitis, and medullary thyroid carcinoma. In this case, the initial ultrasound-guided FNAC was suggestive of a colloid nodule or follicular neoplasm (Bethesda II-IV), and the absence of typical signs such as compressive symptoms or systemic features initially favored a benign etiology. However, the presence of a TI-RADS IV lesion and bilateral necrotic lymphadenopathy raised strong suspicion for malignancy. Tubercular lymphadenitis was ruled out by the absence of systemic signs, lack of caseation on imaging, and no acid-fast bacilli in cytology. Medullary carcinoma was considered but was excluded based on histopathological findings and the absence of amyloid or calcitonin-positive staining. Ultimately, the presence of papillary architecture and characteristic nuclear features on histology confirmed the diagnosis of papillary thyroid carcinoma.

## Conclusions

This case highlights the importance of considering PTC in the differential diagnosis of persistent cervical lymphadenopathy in children, even in the absence of traditional risk factors such as radiation exposure or family history. Early clinical suspicion, targeted imaging, timely surgical intervention, and risk-adapted postoperative management are essential to ensure favorable long-term outcomes in pediatric patients with PTC.
